# A Census of Medicolegal Death Investigation in the United States: A Need to Determine the State of our Nation’s Toxicology Laboratories and Their Preparedness for the Current Drug Overdose Epidemic†, ‡

**DOI:** 10.1111/1556-4029.14277

**Published:** 2020-01-28

**Authors:** Jeri D. Ropero‐Miller, Hope M. Smiley‐McDonald, Stephanie A. Zimmer, Katherine M. Bollinger

**Affiliations:** ^1^ Applied Justice Research Division RTI International 3040 E. Cornwallis Road Research Triangle Park 27709 NC; ^2^ Statistical and Data Sciences Division RTI International 3040 E. Cornwallis Road Research Triangle Park 27709 NC

**Keywords:** forensic toxicology, medicolegal death investigation, overdose, policy, medical examiner, coroner

## Abstract

In 2007, the Bureau of Justice Statistics reported on 2004 data collected from the Census of Medical Examiner and Coroner Offices (CMEC). The CMEC was one of the first comprehensive reports on the state of the medicolegal death investigation system in the United States and included information on administration, expenditure, workload, specialized death investigations, records and evidence retention, and resources. However, the report did not include responses on questions that were related to toxicology such as specimen retention and type of testing. The purpose of this publication is to provide the community with toxicology laboratory‐specific responses from nearly 2000 medical examiner and coroner (MEC) offices. Data obtained from a BJS CMEC public use dataset for any remaining information that was not reported in the 2007 BJS report were evaluated specific to the operation of toxicology laboratories within a MEC office or specific to toxicology testing. The CMEC includes information on average operating budget for MEC offices with internal or external toxicology services, budget for toxicology/microbiology services, respondents’ routine uses of toxicology analysis, toxicology specimen retention time, average turnaround times, use of computerized information management systems, and participation in federal data collections. These historical data begin to address the present state of our nation’s toxicology laboratories within the medicolegal death investigation system and their preparedness for the current drug overdose epidemic.

Annually, the U.S. death toll equates to a little less than 1% of its population. If a death is sudden and unexpected, and the circumstances largely unnatural and unexplained, then it is investigated by a medical examiner’s or coroner’s office to determine its cause and manner as a part of our nation’s public health and safety response. The characteristics of the U.S. medicolegal death investigation (MDI) system are determined at state and local levels, and the system is highly diverse in organization and operation. Approximately half a million deaths are investigated annually 1. The Centers for Disease Control and Prevention (CDC) reported 70,237 overdose mortalities in 2017 (2). Moreover, in 2017, 20 states and the District of Columbia had age‐adjusted drug overdose death rates that were statistically higher than the national rate (2) Figure 1 2, 3, 4 provides the estimated death toll in the United States in 2017 and shows its impact on the MDI system, which has experienced a doubling in overdose investigation since 2007 (36,010 deaths, 1 of 12 investigations) 2, 5.

**Figure 1:**
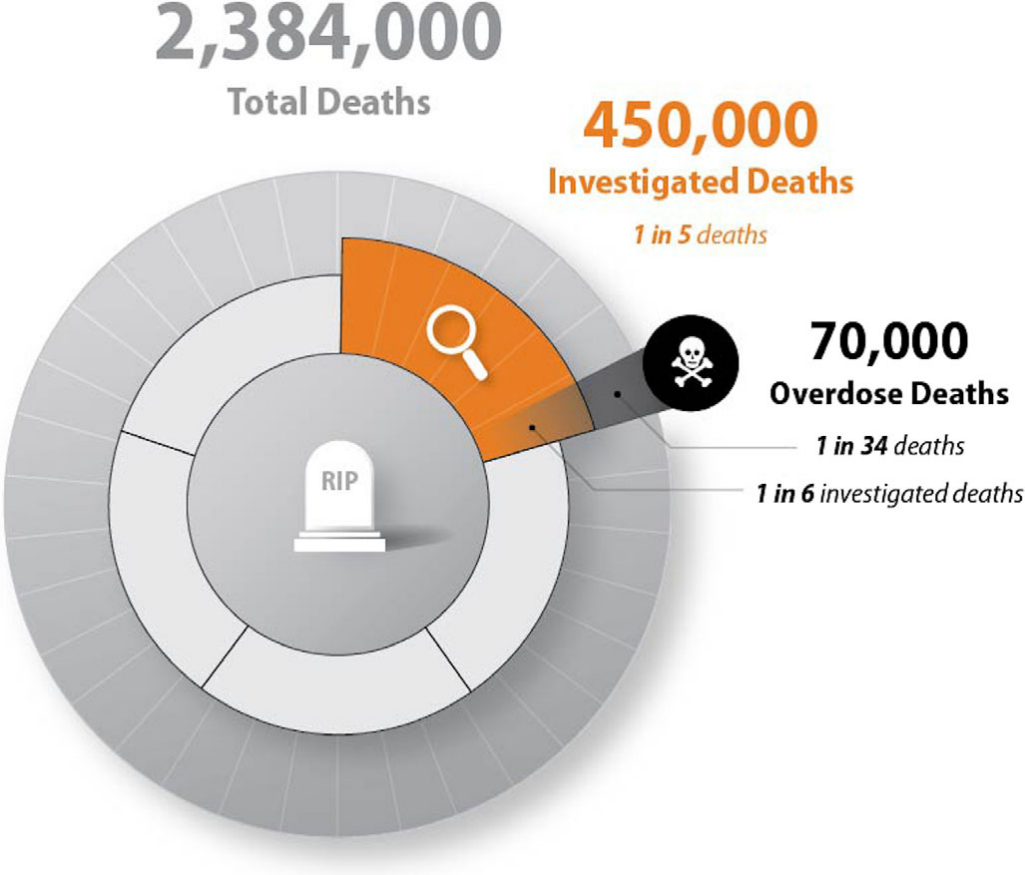
Estimated death toll using 2017 U.S. population and 2017 age‐adjusted death rate 2, 3, 4.

In 2007, the U.S. Bureau of Justice Statistics (BJS) published a Special Report titled *Medical Examiners and Coroners’ Offices, 2004* 1. This report describes the state of the MDI system in the United States and was based on the results of the 2004 BJS Census of Medical Examiner and Coroner Offices (CMEC). The CMEC was sent to almost 2000 medical examiner and coroner (MEC) offices. These offices conduct death scene investigations, perform autopsies, and determine the cause and manner of death.

The 2004 CMEC had an 86% response rate with 1717 of 1998 MEC offices responding to the survey. The CMEC data showed that nearly 1 million deaths were referred to MECs in 2004, which accounted for approximately 40% of all U.S. deaths for that year. The census included a total of 42 questions and sections that inquired about the following areas: administrative information, expenditures, workload, specialized death investigations, records and evidence retention, and resources. A more recent national comparison of MEC offices indicates that the distribution of each type of coroner or medical examiner system (Fig. 2) has changed since the 2007 BJS report, with an increase in the number of coroner‐only states (10 in 2013 vs. 14 in 2004) 3, 6.

**Figure 2:**
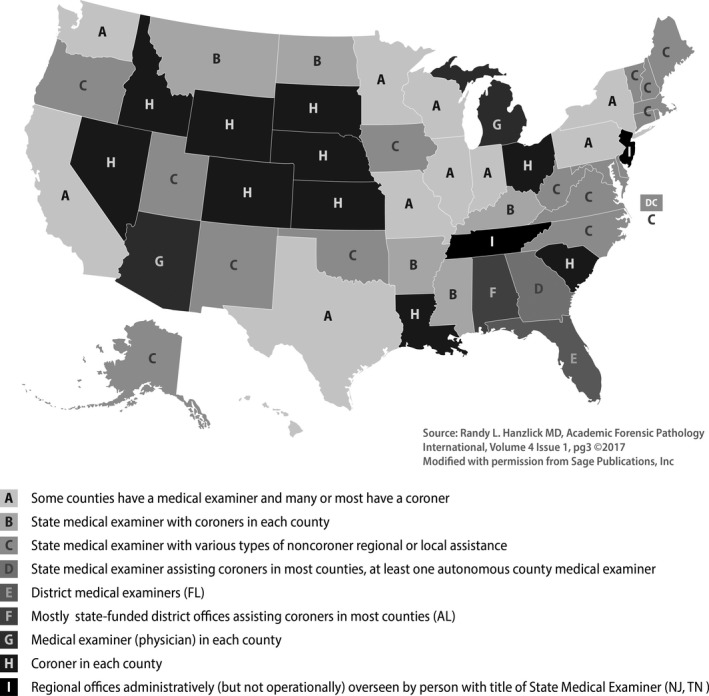
Distribution of medical examiner and coroner offices by state, 2013.

Although the 2007 BJS report presented landmark data about the MDI system, much of the toxicology laboratory operations, casework, and practices were not included. In fact, 6 of the 42 total census questions were specific to the toxicology laboratory. This present analysis uses the 2004 public dataset to detail the toxicological findings reported in the 2004 CMEC census. Another goal of the present publication is to analyze and discuss operational characteristics of forensic toxicology laboratories within U.S. MDI systems.

Our nation faces its worst drug overdose epidemic, which is critically challenging the death investigation system 7, including the management of our toxicology laboratories. This information is being reported now to help inform current data collection efforts, such as the 2018 BJS CMEC Census 6, the Drug Enforcement Administration’s (DEA’s) National Forensic Laboratory Information System (NFLIS), CDC’s Overdose Data to Action (OD2A) 8, and state‐level programs such as prescription drug monitoring programs. Unlike BJS’s other forensic data collection, the Census of Publicly Funded Forensic Crime Laboratories 9, which has been administered about every 5 years, the CMEC was only fielded once prior to 2018. Knowledge of toxicology laboratory operations can provide insight for workload and function, budget and operations, management practices, and testing protocols, which can, in turn, better inform our understanding of the infrastructure in place to respond to the present drug epidemic (and any drug epidemics to follow).

## Methods

### 2004 CMEC Data Collection

RTI International conducted the original data collection for the 2004 CMEC on behalf of BJS (2005‐MU‐MU‐K011) during a 12‐month period beginning in 2005. RTI designed the census questionnaire in coordination with a forensic expert panel review and piloted it to select MEC offices. We used a mixed‐mode data collection approach with mail, email, web, and computer‐assisted telephone interviewing (CATI) options, and thus, responses were received through mail, fax, CATI, and the web‐based instrument. The state of Louisiana was excluded from this census because of the devastation of Hurricane Katrina. The data collected through the census responses represent national estimates. More information about the data collection methodology may be found in the 2007 report 1.

### RTI Data Assessment, 2018

In 2018, RTI evaluated data obtained from a BJS CMEC public use dataset for any remaining information that was not reported in the 2007 BJS report. We evaluated census data specific to the operation of toxicology laboratories within a MEC office or specific to toxicology testing. For respondents completing the surveys, we ran frequencies on each variable to determine patterns in item nonresponse for the items of interest. Overall, the RTI team found the item nonresponse rates to be low. Specifically, RTI calculated the number of toxicology tests requested across MEC offices based on the population served (item response rate 100%), the type of MEC (item response rate 100%), the number of full‐time equivalent (FTE) employees (item response rate 100%) and contractors (item response rate 87%), the operating budget (item response rate 99%), and the type of toxicology testing (routine vs. special request, internal vs. external to MEC office; item response rate 73% and 61%, respectively).

### Analytic Approach

RTI collected data at the MEC office level, but for the present analysis, we further categorized them to whether the MEC offices reported internal toxicology testing (*n* = 92), external toxicology testing (*n* = 935), or both (*n* = 16). Specifically, question C7 on the 2004 CMEC instrument asked respondents 10: *Please indicate whether your office performs the following functions routinely or occasionally/by special request. Please also indicate whether most functions are performed internally (i.e., within your office) or externally (i.e., outsourced to an independent facility such as a health department or commercial laboratory*. Because the cell size was small for those MEC offices that reported both internal and external toxicology testing, those offices were categorized within the “internal” testing category, bringing the total for that category to 108. All data in this analysis are reported as frequencies or percentage frequencies. We used SAS/STAT software, version 9.4 and SUDAAN 11.0.1 to group results by MEC characteristics, workload, function, evidence retention, recordkeeping, and reporting 11, 12.

### Imputations

For the 2004 CMEC data collection, RTI made imputations for missing continuous data elements including FTEs, budgets, and workload items (cases referred and accepted and unidentified decedents handled in an average year). Because the present analysis used the 2004 publicly available dataset the imputations that were calculated for that dataset are relevant; more information about the imputation methodology may be found in the 2007 report 1.

## Results

### MEC Characteristics

Approximately 50% of MEC offices responded to the toxicology testing services questions. The aggregate population for MEC offices reporting offering toxicology services (1043 responses) showed an equivalent distribution of small (42% were <25,000 population served) and medium jurisdictions (43% were 25,000 to 249,999 population served), while the remaining 15% were jurisdictions greater than 250,000. MEC offices reporting toxicology services were distributed as 79% county coroner, followed by 16% county medical examiner offices, 2% district/regional medical examiner offices, and <2% each for the remaining office types (i.e., state medical examiner office, city medical examiner office, district/regional coroner office, and other). These percentages are roughly similar to those reported in BJS’s 2007 report, which found that county coroners’ offices accounted for 80% of all MECs, most of which served small jurisdictions (fewer than 25,000 persons) 1.

Although the 2004 CMEC did not collect FTE and consultant data specific to the toxicology laboratory, some data were obtained. FTE and consultants for “Laboratory support (e.g., technicians/analysts, lab support personnel, toxicologists)” were reported in aggregate (*n* = 1043). The mean of FTE and consultants reported for laboratory support was 0.7 and 0.3, respectively.

The average operating budget for the MEC offices varied based on jurisdiction and population for those MEC offices reporting providing external or internal toxicology testing (Table 1). The average and median annual operating budget was estimated at $553,000 and $49,000, respectively, and accounted for all the toxicology and microbiology testing performed. The annual operating budget included personnel, equipment, supplies, training, accreditation, travel, contractual services, and any other operating costs, but did not include utilities or facilities costs. Across the MEC offices offering toxicology testing, operating budgets were skewed right with the median budget being a little over $48,000 and the median budget for toxicology/microbiology functions being $7000. Of those offices (*n* = 941) that answered the question on toxicology/microbiology budget, 66.5% reported a budget of $0; the $0 values are not included in the calculation of mean and median budgets. Overall, toxicology was the highest ordered procedure, but represented a small proportion of the annual operating budget. Basic toxicology testing costs hundreds of dollars and can increase to thousands of dollars in complicated cases requiring expanded test panels and specialty testing 13, 14, 15. When an operating budget is set at the beginning of a fiscal year, toxicology testing needs cannot be readily predicted, and budget shortages may exist. Notably, the 2007 report produced by BJS found that across all respondents (*N* = 1998), the average budget per office was $387,000 and the median budget was $37,000 1.

**Table 1:** Average and median operating budget by total offices offering toxicology services and by toxicology/microbiology services offered, 2004.

	Average	Median
Total MEC offices offering internal or external toxicology services (*n* = 995)	$553,00	$48,000
Toxicology/microbiology (*n* = 315)	$49,000	$7,000

Does not include offices reporting zero annual budget.

Table 2 breaks down the average number of accepted cases by the requested procedure. MECs that provided toxicology services requested an average of 184.8 toxicology analyses in 2004, which was the highest ordered procedure. The next most commonly ordered procedure was a review of medical records from a healthcare provider with an average of 169.7 cases, followed by death scene investigations (168.4 average cases). Thus, in 2004, toxicology testing contributed significantly to the workload and function of MEC offices.

**Table 2:** Average number of accepted cases by procedure performed.

Procedure performed	MEC Offices Offering Toxicology Services (*N* = 1043)
	Average
Toxicology analysis	184.8
Review of medical records from healthcare provider	169.7
Death scene investigation	168.4
Complete autopsy	135.6
Radiology	56.3
Microbiology	36.7

Moreover, CMEC respondents were asked the extent to which their office performed several types of functions routinely or occasionally/by special request. Toxicology analysis was performed routinely in 58% (811 of 1043 respondents). Although the present analyses represent only those MEC offices that offered toxicology services (internally or externally), this percentage is similar to the 2007 report findings, which showed that 51% of accepted cases received toxicology analysis overall, and among large jurisdictions serving 250,000 populations or larger, it was 57% 1. Notably, the question allowed for general responses and did not identify if the special requests were for specific analytes (i.e., analytes not tested because of infrequency, nonvalidated methods, reference standard unavailable to laboratory). Of the 811 respondents reporting if their toxicology testing was performed internally, externally, or a combination of both, 8 of 10 MEC offices (83.7%) performed toxicology testing externally, meaning they use a reference laboratory and do not perform testing in‐house. The wording of the question could not rule out if an office was responding to all toxicology testing for its laboratory or a subset of cases that required sending to a reference laboratory. Because 85% of MEC offices served a jurisdiction of fewer than 250,000, resources to support in‐house toxicology testing would not be expected.

The average turnaround time in days for case completion by MEC offices reporting on toxicology services was less than 2 months, with the least time required for natural deaths (9.6 days) and longer times required in undetermined (47.4 days) and homicide (34.4 days) cases. As reported, these data do not specifically indicate that these are drug‐related cases. Personnel (46%), equipment (44%), and training (35%) were the most often identified resources needed to improve turnaround time.

The 2004 census also captured information concerning evidence retention, recordkeeping, and reporting associated with toxicology services in MEC offices. As shown in Table 3, over half of MEC offices (54%) reported “no established period” for toxicology specimen retention, although 28% retained specimens for 10 months or more, and 17% retained specimens for 6 months or less.

**Table 3:** Medical examiner and coroner offices by toxicology laboratory services and evidence retention, information system infrastructure, 2004.

Characteristic	Internal Toxicology Testing (*n* = 108)		External Toxicology Testing (*n* = 935)		All MEC Offices Offering Toxicology Services (*n* = 1043)	
	Number	Percent	Number	Percent	Number	Percent
Retention of toxicology specimens						
0–3 months	9	9.5	58	7.8	67	8.0
4–6 months	11	11.6	66	8.9	77	9.2
7–9 months	0	0.0	5	0.7	5	0.6
10–12 months	25	26.3	106	14.2	131	15.6
More than 12 months	20	21.1	86	11.6	106	12.6
Indefinite	30	31.6	423	56.9	453	54.0
Access to the internet						
Yes	90	84.1	777	83.9	867	83.9
Computerized information management system						
Yes	59	55.1	303	32.8	362	35.1
Yes–centralized system	55	53.4	229	28.1	284	31.0

Additionally, during 2004, less than 10% of MEC offices reported to federal data collection efforts. Examples specific to toxicology and drug‐related data include 10% of MEC offices reporting to the CDC National Violent Death Reporting System, 7% reporting to the Department of Health and Human Services’ Drug Abuse Warning Network (no longer operational), and 6% reporting to other federal data collection efforts. In 2004, participation in national federal data collections was hindered by resource limitations (53%), unavailable personnel (48%), and lack of resources for data conversion to other systems (45%) (data not shown).

Use of computerized systems to link data, improve accessibility, and manage data and reporting of results is also of interest for toxicology services within MEC offices. In this analysis, 84% of MEC offices reporting toxicology services used the internet as part of their information system infrastructure in 2004, and 35% used a computerized information system. In almost all cases, if a MEC office had a computerized information system, it was centralized (Table 3).

## Discussion

This study analyzed publicly accessible BJS 2004 data to report on the toxicology‐related findings and characteristics that provide insight and context for personnel, budgets, and workload across a national census of nearly 2000 MEC offices. Interest in the current state of the MDI system within the United States has increased in the past several years. Notably, the past 3‐year National Archive of Criminal Justice Data usage data (from October 2016‐October 2019) show that the 2004 CMEC public dataset has been downloaded nearly 240 times across 73 unique users 3, 16. Moreover, a 2016 report published by the Office of Science and Technology Council’s (OSTC’s) 7 Fast‐Track Action Committee on Strengthening the medicolegal death investigation system discussed needs specific to postmortem toxicology testing and made policy recommendations to address issues related to accessing and working with MEC data. The report encouraged nationwide coordination to assess current needs and opportunities “to support high‐quality postmortem toxicology testing among decedents with possible exposure to drugs, chemicals, and other toxins in the workplace, home, environment, and transportation sector.” As stated in the 2016 OSTC report, the minimal participation of MEC offices in federal data collection efforts will negatively impact the MDI data systems and impair the nation’s ability to address the public safety threats. The Organizational of Scientific Area Committees (OSAC) formed by the National Institute of Standards and Technology and the National Institute of Justice, Department of Justice have worked to identify existing high‐quality standards in discipline specific topics, including medicolegal death investigation and toxicology, to facilitate the development of new standards by standards development organizations such as the AAFS Academy Standards Board. To date, there are 19 standards approved for the OSAC Registry; two of which are specific to toxicology 17.

The OSTC report also called for more forensic toxicologists and broader access to affordable and comprehensive toxicological tests. The extent of drug involvement (e.g., unintentional injuries, suicides, homicides) in investigation of violent deaths is difficult to enumerate and recognize within the current MDI data collection systems available at local, state, and national levels. This knowledge and improved understanding play “a crucial role in support of global health security by protecting public health and safety and combating emerging threats” 7. Similarly, a September 2019 legislative hearing of the U.S. House of Representatives Committee on Science, Space, and Technology titled “Raising the Bar: Progress and Future Needs in Forensic Science” reiterated needs in forensic science to include requirements for standards of practice, professional certification, staffing enhancements, and workforce resiliency in MDI 18.

In the present analyses, we also found that nearly half of MEC offices reported “no established period” for toxicology specimen retention. Not having an evidence retention policy for toxicology services can negatively impact the accuracy of reporting drug‐related deaths. For example, novel psychoactive substances are known to rapidly emerge and wane regionally, sometimes within months. If specimens are inadequately retained, these drug deaths can go unproven and undocumented when the specimens are discarded and unavailable for testing for an unknown substance once its threat is identified and questioned. A minimum time of storing specimens of 6 months or less is equally of concern.

The present study documents the earliest collection of data characterizing the toxicology testing performed within the MEC offices in the United States. Notably, there were more than 1000 responses specific to toxicology laboratory services. Data were collected at the MEC office level and analyzed and presented in the context of MEC offices reporting internal toxicology testing, external toxicology testing, or overall toxicology analysis (i.e., combined internal and external toxicology testing). This analysis is limited because many CMEC questions were not asked specific to toxicology analysis, and thus, the true measurement error could not be determined. Hence, all data are reported as frequency of reporting and percentage frequencies (Table S1).

Although this is the first census of medicolegal death investigation in the United States to yield a high response rate, the original report did not focus on the toxicology testing performed in these MEC offices. The information collected from this study helps us to understand how the data from the drug‐related mortality and toxicology cases were collected, analyzed, and reported during a 1‐year period. This study aimed to inform current data collection efforts about the toxicology section of MEC systems to improve suitability (timeliness), uniformity, accessibility, quality, and utility of MDI data. Although these data represent 2004 findings, continued and expanded data collection efforts are warranted to determine the state of our nation’s postmortem toxicology laboratories and preparedness for current and future drug overdose epidemics.

To that end, BJS is presently completing the 2018 CMEC that will include such measures (://www.bjs.gov/content/pub/pdf/cmecosol.pdf) and provide needed comparisons to the 2004 census. In addition, DEA has recently published reports from its recent surveys of MEC offices and toxicology laboratories under the NFLIS program that also address some of the issues raised herein (://www.nflis.deadiversion.usdoj.gov/reports.aspx). For example, the findings in this report showed that a little over one‐third of MECs had an information management system in 2004. The 2017 NFLIS survey showed that, of responding MECs, 32% had computerized networked systems, 30% had partially computerized systems with some manual recordkeeping, and 31% had a manual recordkeeping system 2. The stark reality that a substantial proportion of the national MDI system lacks basic information infrastructure will challenge federal efforts to obtain national, systematic data from MECs.

Given that MEC offices investigate nearly 450,000 deaths annually and recent CDC reports indicate that over 70,000 Americans died from a drug overdose in 2017, the nation’s drug epidemic is contributing to one of every six deaths investigated by MEC and requires toxicology testing. This is a critical and crippling impact. This study indicates that toxicology testing within MEC offices had many challenges 15 years ago. Current drug overdose statistics suggest that MECs are facing overwhelming caseloads that require more complex and expensive investigations. Notably, recent data from DEA’s NFLIS‐MEC program suggest that as part of their accepted caseload, an average of 46% of MECs performed toxicology testing 2, and drugs such as alcohol, amphetamines, cocaine, and opiates or opioids other than heroin or fentanyl tested in 75% of cases submitted for toxicology. Data such as those from the CMEC and NFLIS programs can help to define epidemiology of drug use, data trends, and emergence of new substances; inform current data collection trends at federal, state, and local levels and advise public health and safety programs including those addressing overdose preventions and deaths. This study reports on the history and begins to address the present state of our nation’s toxicology laboratories within the MDI system and their preparedness for the current drug overdose epidemic.

## Supporting information


**Table S1.** Medical examiner and coroner offices by toxicology laboratory services and selected characteristics, 2004.
