# Supplementary material for: A Census of Medicolegal Death Investigation in the United States: A Need to Determine the State of our Nation’s Toxicology Laboratories and Their Preparedness for the Current Drug Overdose Epidemic
Source: J Forensic Sci. 2020 Jan 28;65(2):544–9. doi: 10.1111/1556-4029.14277 (PMC7065112; doi:10.1111/1556-4029.14277)
Supplement: Supplementary file 1 — Table S1. Medical examiner and coroner offices by toxicology laboratory services and selected characteristics, 2004. [file JFO-65-544-s001.docx]

SUPPLEMENTAL TABLE 1—*Medical examiner and coroner offices by toxicology laboratory services and selected characteristics, 2004.*

| Characteristic | | Toxicology Testing | | | | | |
| --- | --- | --- | --- | --- | --- | --- | --- |
|  |  | Internal Toxicology Testing (n=108) | | External Toxicology Testing (n=935) | | All With Toxicology Testing (n=1043) | |
|  |  | Number | Percent | Number | Percent | Number | Percent |
| Population Served (A5): Enter the aggregate population of the jurisdiction(s) your office serves | | | | | | | |
|  | 1,000,000 or more | 29 | 26.9 | 23 | 2.5 | 52 | 5.0 |
|  | 500,000 to 999,999 | 15 | 13.9 | 34 | 3.6 | 49 | 4.7 |
|  | 250,000 to 499,999 | 7 | 6.5 | 45 | 4.8 | 52 | 5.0 |
|  | 100,000 to 249,999 | 7 | 6.5 | 113 | 12.1 | 120 | 11.5 |
|  | 50,000 to 99,999 | 6 | 5.6 | 115 | 12.3 | 121 | 11.6 |
|  | 49,999 or less | 44 | 40.7 | 605 | 64.7 | 649 | 62.2 |
| Population Served (A5): Enter the aggregate population of the jurisdiction(s) your office serves | | | | | | | |
|  | Large jurisdiction (250,000 or more) | 51 | 47.2 | 102 | 10.9 | 153 | 14.7 |
|  | Medium jurisdiction (25,000 to 249,999) | 31 | 28.7 | 421 | 45.0 | 452 | 43.3 |
|  | Small jurisdiction (Less than 25,000) | 26 | 24.1 | 412 | 44.1 | 438 | 42.0 |
| Type of MEC (A3): Which of the following best describes your office? Mark only one. | | | | | | | |
|  | State medical examiner office | 7 | 6.5 | 11 | 1.2 | 18 | 1.7 |
|  | District/regional medical examiner office | 9 | 8.3 | 16 | 1.7 | 25 | 2.4 |
|  | County medical examiner office | 29 | 26.9 | 135 | 14.4 | 164 | 15.7 |
|  | City medical examiner office | 1 | 0.9 | 1 | 0.1 | 2 | 0.2 |
|  | District/regional coroner office | 3 | 2.8 | 7 | 0.7 | 10 | 1.0 |
|  | County coroner office | 59 | 54.6 | 764 | 81.7 | 823 | 78.9 |
|  | Other | 0 | 0.0 | 1 | 0.1 | 1 | 0.1 |
| Number of FTE positions for your office (A8): Enter the number of FTE and consultant/contractor positions for your office in 2004 that fall into the following categories | | | | | | | |
|  | Forensic pathologists | 3.0 | 2.0 | 0.7 | 0.0 | 1.0 | 0.0 |
|  | Other medical examiners and/or coroners | 3.1 | 0.0 | 1.9 | 0.0 | 2.0 | 0.0 |
|  | Ancillary death investigation personnel | 9.0 | 4.0 | 1.5 | 0.0 | 2.3 | 0.0 |
|  | Forensic specialists | 0.4 | 0.0 | 0.0 | 0.0 | 0.1 | 0.0 |
|  | Laboratory support | 5.2 | 1.0 | 0.1 | 0.0 | 0.7 | 0.0 |
|  | Computer specialists and IT support | 0.4 | 0.0 | 0.1 | 0.0 | 0.1 | 0.0 |
|  | Administrative | 5.3 | 2.8 | 0.9 | 0.0 | 1.4 | 0.0 |
|  | Other | 2.7 | 0.0 | 0.4 | 0.0 | 0.6 | 0.0 |
| Number of consultant/contractor positions for your office (A8) | | | | | | | |
|  | Forensic pathologists | 0.3 | 0.0 | 0.6 | 0.0 | 0.5 | 0.0 |
|  | Other medical examiners and/or coroners | 0.5 | 0.0 | 0.7 | 0.0 | 0.7 | 0.0 |
|  | Ancillary death investigation personnel | 1.9 | 0.0 | 0.5 | 0.0 | 0.6 | 0.0 |
|  | Forensic specialists | 1.1 | 0.0 | 0.3 | 0.0 | 0.4 | 0.0 |
|  | Laboratory support | 0.3 | 0.0 | 0.3 | 0.0 | 0.3 | 0.0 |
|  | Computer specialists and IT support | 0.2 | 0.0 | 0.1 | 0.0 | 0.1 | 0.0 |
|  | Administrative | 0.6 | 0.0 | 0.1 | 0.0 | 0.1 | 0.0 |
|  | Other | 0.1 | 0.0 | 0.1 | 0.0 | 0.1 | 0.0 |
| Resources needed to improve turnaround time (F1): Which of the following additional resources would be necessary to improve your overall turnaround time for case completion? Mark all that apply. | | | | | | | |
|  | Personnel | 69 | 67.0 | 400 | 43.9 | 469 | 46.3 |
|  | Training | 35 | 34.0 | 321 | 35.2 | 356 | 35.1 |
|  | Laboratory/facility space | 39 | 37.9 | 246 | 27.0 | 285 | 28.1 |
|  | Administrative and evidence storage facilities | 31 | 30.1 | 211 | 23.2 | 242 | 23.9 |
|  | Equipment | 59 | 57.3 | 389 | 42.7 | 448 | 44.2 |
|  | Analytical instrumentation and laboratory supplies | 33 | 32.0 | 146 | 16.0 | 179 | 17.7 |
|  | Other | 13 | 12.6 | 123 | 13.5 | 136 | 13.4 |
|  | None of the above | 13 | 12.6 | 258 | 28.3 | 271 | 26.7 |
| Federal data collection efforts (F3): In which federal data collection efforts does your office currently participate? Mark all that apply. | | | | | | | |
|  | National Violent Death Reporting System (NVDRS) | 11 | 11.5 | 90 | 10.1 | 101 | 10.3 |
|  | Drug Abuse Warning Network (DAWN) | 21 | 21.9 | 55 | 6.2 | 76 | 7.7 |
|  | Other | 10 | 10.4 | 48 | 5.4 | 58 | 5.9 |
|  | None of the above | 66 | 68.8 | 738 | 83.1 | 804 | 81.7 |
| Barriers to participating in federal data collection efforts (F4): What are the main potential barriers for your office to participate in federal data collection efforts? | | | | | | | |
|  | Lack of electronic records | 37 | 36.3 | 384 | 42.7 | 421 | 42.1 |
|  | Lack of resources for data conversion to other systems | 53 | 52.0 | 401 | 44.6 | 454 | 45.4 |
|  | Concerns about privacy | 15 | 14.7 | 130 | 14.5 | 145 | 14.5 |
|  | Unavailable personnel | 65 | 63.7 | 419 | 46.6 | 484 | 48.4 |
|  | Unwillingness to share data with federal agencies | 2 | 2.0 | 14 | 1.6 | 16 | 1.6 |
|  | Redundancy of federal data requests from multiple agencies | 12 | 11.8 | 100 | 11.1 | 112 | 11.2 |
|  | Resource limitations | 51 | 50.0 | 478 | 53.2 | 529 | 52.8 |
|  | Concerns that the effort will not benefit my jurisdiction | 15 | 14.7 | 162 | 18.0 | 177 | 17.7 |
|  | *Other barriers* | 13 | 12.7 | 90 | 10.0 | 103 | 10.3 |
|  | *None of the above* | 19 | 18.6 | 199 | 22.1 | 218 | 21.8 |

|  |
| --- |
| Budget (B2): Enter your office's total operating budget for the 2004 calendar or fiscal year. Include personnel, equipment, supplies, training, accreditation, travel, contractual services, and any other operating costs. Do not include utilities or facilities costs.  (B3): Enter approximate budgetary amounts dedicated to each of the following areas during the 2004 calendar or fiscal year. Include capital purchase and maintenance costs. The budget amounts should sum to the total provided in question B2. |

| Characteristic | Internal Toxicology Testing (n=108) | | External Toxicology Testing (n=935) | | All With Toxicology Testing (n=1043) | |
| --- | --- | --- | --- | --- | --- | --- |
|  | Mean | Median | Mean | Median | Mean | Median |
| Total ME/C Office | 1,943,629 | 345,741 | 364,396 | 40,000 | 528,236 | 42,444 |
| Toxicology/Microbiology | 84200.1 | 1000.0 | 9210.5 | 0.0 | 16462.4 | 0.0 |
